# Prone positioning in cardiac surgery: for many, but not for everyone

**DOI:** 10.1186/2197-425X-3-S1-A103

**Published:** 2015-10-01

**Authors:** C Lim, M Wenzl, AM Dell´Aquila, A Junger, T Fischlein, G Santarpino

**Affiliations:** Klinikum Nürnberg - Paracelcus Medical University, Cardiac Surgery - Cardiovascular Center, Nuremberg, Germany; Klinikum Nürnberg - Paracelcus Medical University, Anesthesiology, Nuremberg Germany; University Hospital Münster, Cardiac Surgery, Münster Germany

## Introduction

Prone positioning is a therapeutic maneuver to improve arterial oxygenation in patients with acute lung injury that is not implemented in most centers performing adult cardiac surgery. The aim of this study was to review our experience with prone positioning, which has become increasingly used at our center over the last years.

## Methods

From 2010 to 2014, 127 adult patients with postoperative acute respiratory failure were treated with prone positioning in addition to supportive therapy. Preoperative and intensive care unit data obtained from medical records were retrospectively reviewed. Univariate and multivariate logistic regression analyses were performed to identify independent risk factors associated with in-hospital mortality.

## Results

In-hospital mortality was 22.8% (n = 29). No complications related to the proning maneuver were recorded. No significant differences were observed in preoperative risk factors between patients who survived (group S) and those who died (group D), except for age (62.7 ± 11.2 vs 70.2 ± 11.3; p = 0.007) and EuroSCORE II (6.57 ± 8.26 vs 9.22 ± 6.99; p = 0.07). Age retained statistical significance at multivariate analysis (p = 0.03, OR 1.07). Pre-pronation values of the PaO_2_/FiO_2_ ratio were significantly different between groups (group S vs D: 150 ± 56 vs 115 ± 46; p = 0.006), but only pre-pronation FiO_2_ remained highly significant at multivariate analysis (0.67 ± 0.16 vs 0.82 ± 0.18; univariate p = 0.0006, multivariate p = 0.001, OR 6.11). Patients who died showed a higher improvement of the PaO_2_/FiO_2_ ratio immediately after pronation compared with survivors (group S vs D: 219 ± 90 vs 207 ± 100, p = 0.56; at within group analysis between pre-pronation and 1 hour after prone positioning: group S p = 0.49, group D p = 0.019; at 12 hours: 286 ± 123 vs 240 ± 120, p = 0.06; at within group analysis between 1 hour and 12 hours after prone positioning: group S p = 0.15; group D p = 0.17; between groups p = 0.05). Patients who died had higher pea k white blood cell counts than survivors (group S vs D: 17.67 ± 5.95 vs 26.07 ± 9.78 × 10^3^/ml; p = 0.0001) and a higher rate of low output syndrome prior to prone positioning (group S vs D: 9 [9.18%] vs 15 [51.72%] patients; p = 0.0001). Both variables were found to be independent predictors of mortality at multivariate analysis (white blood cell count: p = 0.005, OR 1.11; low output syndrome: p = 0.0002, OR 20.5).

## Conclusions

Prone positioning is a safe treatment option for adult patients with postoperative acute respiratory failure. Its efficacy seems to be closely related to two factors: the optimal timing of application of the procedure and a noncardiogenic etiology as the cause of low PaO_2_/FiO_2_ ratio.

